# Epidemiology and patterns of musculoskeletal motorcycle injuries in the USA

**DOI:** 10.12688/f1000research.4995.1

**Published:** 2015-05-12

**Authors:** Sean T. Burns, Zbigniew Gugala, Carlos J. Jimenez, William J. Mileski, Ronald W. Lindsey

**Affiliations:** 1 Department of Orthopaedic Surgery and Rehabilitation, The University of Texas Medical Branch, Galveston, TX, TX 77555, USA; 2 Department of Surgery, The University of Texas Medical Branch, Galveston, TX, TX 77555, USA

**Keywords:** musculoskeletal motorcycle injuries, epidemiology, fractures

## Abstract

**Introduction**: Motorcycles have become an increasingly popular mode of transportation despite their association with a greater risk for injury compared with automobiles. Whereas the recent incidence of annual passenger vehicle fatalities in the United States of America (USA) has progressively declined, motorcycle fatalities have steadily increased in the past 11 years. Although motorcycle injuries (MIs) have been studied, to the author’s knowledge there are no published reports on MIs in the USA during this 11-year period.

**Methods** *:* Study data were derived from a prospectively collected Level I trauma center database. Data sampling included motorcycle crash injury evaluations for the 10-year period ending on 31 August 2008. This retrospective analysis included patient demographic and medical data, helmet use, Glasgow coma scale (GCS) score, injury severity score (ISS), length of hospital stay (LOS), specific injury diagnosis, and death. Data statistics were analyzed using the Spearman correlation coefficient, Kruskal-Wallis tests, and logistic regression.

**Results**: The study identified 1252 motorcycle crash injuries. Helmets were worn by 40.7% of patients for which helmet data were available. The rates of the most common orthopedic injuries were tibia/fibula (19.01%), spine (16.21%), and forearm (10.14%) fractures. The most common non-orthopedic motorcycle crash injuries were concussions (21.09%), skull fractures (8.23%), face fractures (13.66%), and hemo- and pneumothorax (8.79%). There was a significant correlation between greater age and higher ISS (r=0.21, P<0.0001) and longer LOS (r=0.22, P<0.0001). Older patients were also less likely to wear a helmet (OR=0.99, 95% CI: 0.98, 0.997), associated with a significantly higher risk for death (after adjustment for helmet use OR=1.03, 95% CI: 1.00, 1.05). All patients without helmets had a significantly lower GCS score (P=0.0001) and a higher mortality rate (after adjustment for patient demographic data OR=2.28, 95% CI: 1.13, 4.58).

**Conclusion** *:* Compared with historical reports, the prevalence of skull, face, spine, and pelvis fractures have increased in American motorcycle crashes. Compared to recent European studies, the incidence of USA skull and face fractures is much higher, while the incidence of USA spine and pelvis fractures is more comparable; however, this is not associated with increased in-hospital mortality.

## Introduction

Injury secondary to motorcycle crashes is a major problem in the United States of America (USA). In 2007, the number of registered motorcycles in the USA totaled 7,138,476, of which 387,915 were registered in Texas ^19, 21^. That same year, 103,000 Americans were injured in motorcycle crashes, with 5,154 of them killed. Texas ranked third in terms of fatalities behind Florida and California ^18^.

In recent years, the annual incidence of automobile-related fatalities in the USA has progressively decreased, while the annual incidence of motorcycle crash fatalities has progressively increased ^19^. However, despite the expanding use of motorcycles, there has been a paucity of orthopedic epidemiological studies related to motorcycle crash in recent years. Our institution has been the primary Level I trauma facility for Galveston County and the surrounding region. The area is one of the most popular motorcycle destinations in Texas, and Galveston is home to the state’s largest motorcycle rally ^1^. The purpose of this study was to review a single-institution experience with MIs in regard to the epidemiology and patterns of both musculoskeletal and non-musculoskeletal injuries. In addition, this study will compare our modern USA motorcycle injury data with those of earlier international reviews ^2, 27^.

## Materials and methods

The study was conducted in compliance with the University of Texas Medical Branch policies and regulations regarding human subject research following study protocol review and approval by the Institutional Review Board (approval IRB #09-259). Study data were derived from a prospectively collected injury database at our Level I trauma center. The data sampling included 1,221 patients injured in motorcycle crashes for the 10-year period ending August 31, 2008. Each motorcycle trauma event was considered separately and evaluated independently; patients with more than one motorcycle trauma event evaluation during the study time period had each event considered as a separate incident.

This retrospective analysis included data on patient age, gender, race/ethnicity, documentation of helmet use, Glasgow Coma Scale (GCS) score at the time of presentation in the emergency department, injury severity score (ISS), length of hospital stay (LOS), specific injury diagnosis, and mortality.

Descriptive statistics were used to describe the sample characteristics (age, gender, race) and distribution of the outcome variables (GCS, ISS, LOS, type of injury, and death). Non-parametric methods (Spearman correlation coefficient and Kruskal-Wallis tests) were used to test the association between the general patient demographic data, helmet use, and the outcome variables ISS and LOS. Logistic regression analyses were used to test the association of the general patient demographic data and helmet use with patient GCS classification (mild vs. moderate/severe), vital status at discharge and type of injuries (with/without central nervous system injury, dislocation, fracture, and thorax injury). All tests were two-sided with alpha of 0.05 and were performed using SAS 9.3 (SAS Institute Inc, Cary, NC, USA).

## Results

### Demographic and outcome data

The study identified 1,252 motorcycle trauma event evaluations performed at our institution within a 10 year period. Of these trauma event evaluations, 31 were for a patient’s second or third motorcycle injury during the study period. These multiple-event evaluations involved 28 patients and constituted 59 of all 1,252 evaluations. Twenty-five patients were evaluated twice and three patients were evaluated three times. The interval between subsequent trauma event evaluations among these 28 patients ranged from 2 days to 8.5 years.

Males made up 83% of the motorcycle injury evaluations. The average patient age was 36.0 years (range 4–83 years). There was an 8:1:1 White:Black:Hispanic ratio ( Table I). Helmet use could be established for 1093 (87.3%) of motorcycle event evaluations. Within that subset, helmets were worn in 445 cases (40.7%) and not worn in 648 cases (59.3%).

**Table I.** Patient Characteristics ^a^.

Descriptive	Mean (SD)	%
Age, years	36.0 (13.7)	
Sex		
Male		83
Female		17
Race		
White		78.7
Black		10.3
Hispanic		10.2
Other		0.8	
Helmet		
Yes		35.5	
No		51.8
Unknown		12.7	
Outcomes		
GCS		
Minor		90.02	
Moderate		1.04
Severe		8.31	
Not available		0.64	
ISS	9.4 (11.0)	
LOS, days	4.4 (8.9)	
Death		
Alive		95.6	
Dead		4.4 ^a^

^a^ All values are for number of evaluations ( *N* = 1252).

The average GCS score at presentation for the 1,244 trauma event evaluations with available data was 13.9. The diagnosis was a minor brain injury (GCS 13–15) in 1127 (90.6%), a moderate brain injury (GCS 9–12) in 13 (1%), and a severe brain injury (GCS 3–8) in 104 (8.4%).

The average ISS for all evaluations was 9.4 (median 5, range 0–75). The mortality rate (at arrival and/or post-admission) was 4.4% (55 evaluations), with 40% (22) of those patients dead on arrival (DOA). Excluding the DOA cases, the mortality rate in all other (in-hospital) evaluations fell to 2.7% (33/1230). The average LOS was 4.4 days (median 1 day, range 0–121 days).

### Specific injury data

The incidence and specific types of motorcycle injuries are listed in Table II– Table V. The most common orthopedic motorcycle injuries were tibia/fibula, spine, and forearm fractures, which occurred in 238 (19%) evaluations, 203 (16.2%), and 127 (10.1%) respectively ( Table II and Table III). The highest rates of non-orthopedic motorcycle injuries were for concussions (264 evaluations, 21.09%), skull fractures (103, 8.23%), face fractures (171, 13.66%), and hemo- and pneumothorax injuries (110, 8.79%) ( Table IV). Abdominal injuries (excluding vessel injuries) were seen in 192 evaluations (15.3%) ( Table V).

**Table II.** Fractures.

Bone	Fractures ^a^	Open ^b^	Percent of patients ^c^	Percent of fractures that were open ^d^
Skull	103	16	8.23	15.53
Face	171	3	13.66	1.75
Spine Cervical	60		4.79	
Thoracic	48		3.83	
Lumbar/Sacrum	95		7.59	
				
Ribs/Sternum	212		16.93	
Clavicle	110	3	8.79	2.73
Scapula	54		4.31	
Humerus	39	9	3.12	23.08
Radius/Ulna	127	26	10.14	20.47
Hand/Wrist	108	9	8.63	8.33
				
Acetabulum	34	1	2.72	2.94
Pelvis	76	5	6.07	6.58
Femur	99	19	7.91	19.19
Patella	10	4	0.80	40.00
Tibia/Fibula	238	83	19.01	34.87
Foot	108	16	8.63	14.81
Total	1692			

^a^ For patients with bilateral fractures only one side was counted (i.e., bilateral fractures were counted as one fracture not two). ^b^ Open fractures are included in the overall fractures column. ^c^ The percentages listed in this column are the percentages of patients with each fracture, as opposed to the percentages of fractures that are of a particular type (e.g., there was a skull fracture in 8.23% of evaluations, where skull fractures were 6.09% of all fractures). ^d^ The percentages listed in this column are the percentages of fractures that were open (e.g., 40% of patella fractures were open, but open patella fractures occurred in only 0.32% of evaluations).

**Table III.** Dislocations.

Joints	Closed dislocations	Open dislocations	Percent of patients ^a^	Percent of fractures that were open ^b^
Jaw	1	0	0.08	0.00
Shoulder	32	0	2.56	0.00
Elbow	4	1	0.32	25.00
Wrist	12	0	0.96	0.00
Finger	5	1	0.40	20.00
Hip	8	0	0.64	0.00
Knee	9	1	0.72	11.11
Ankle	9	4	0.72	44.44
Foot	3	1	0.24	33.33

^a^ Evaluations in which there were bilateral dislocations were counted as having one. ^b^ The percentages listed in this column are the percentages of evaluations with each dislocation, as opposed to the percentage of dislocations that are of a particular type. ^c^ The percentages listed in this column are the percentages of dislocations that were open.

**Table IV.** Central nervous system injuries.

Injury	Number	Percent ^a^
Concussion	264	21.09
Contusion	63	5.03
Subarachnoid hemorrhage	37	2.96
Subdural hemorrhage	26	2.08
Extradural hemorrhage	11	0.88
Other intracranial injury	23	1.84
Central cord syndrome	3	0.24
Cord injury with fracture	16	1.28
Cord injury without bone involvement	7	0.56
Total	450	

^a^ The percentages listed are for the rates of particular injuries in the motorcycle injury evaluations.

**Table V.** Thorax, abdomen, and pelvis injuries.

Injury	No.	% ^a^
Hemo- and pneumothorax	110	8.79
Heart	9	0.72
Lung	114	9.07
Other chest ^b^	15	1.19
Liver	46	3.66
Gastrointestinal	25	1.99
Spleen	61	4.85
Urogenital	33	2.63
Other abdomen ^b^	27	2.15
Total	440	

^a^ The percentages listed are for the rates of particular injuries in the motorcycle injury evaluations. ^b^ Vessel injuries are not included.

### Data analysis results

Older patients were less likely to use a helmet (OR=0.99, 95% CI: 0.98, 0.997), which was associated with a significantly higher risk for death (after adjustment for helmet use OR=1.03, 95% CI: 1.00, 1.05). Older patients without helmets had a significantly lower GCS score (P=0.0001) and higher mortality rate (after adjustment for patient demographic characteristics OR=2.28, 95% CI: 1.13, 4.58).

Logistic regression analysis demonstrated that greater age was significantly associated with more severe injury (Spearman correlation coefficient r=0.21, P<0.0001) and a longer hospital stay (Spearman correlation coefficient r=0.22, P<0.0001). Greater age was also associated with significantly higher risks for death following injury, for fracture, and for thorax injury.

Whites had significantly higher ISS and LOS and higher risk for fracture.

Among all patients who did not wear a helmet, the risk was significantly higher for a more severe GCS score (adjusted odds ratio 2.98) and for death (adjusted odds ratio 2.95). These patients were also more likely to have a fracture of the skull (P<0.001) or face (P<0.001).

Fracture results from our current study and from earlier studies from England, Sweden, and the USA are presented in Table VI. The rates of skull and face fractures are higher in our current study than in England or Sweden. The incidence of skull, spine, and rib fractures has nearly doubled in the USA over the last 30 years, and pelvis fractures are much more prevalent.

**Table VI.** Fracture Literature Comparisons.

			Yorkshire region, England		Sweden			Fresno County, California, USA		Oakland, California (military base), USA		Sacramento County, California, USA	
	Present study		Ankarath *et al.* ^2^		Wladis *et al.* ^27^		Zettas *et al.* ^28^		Denaner *et al.* ^9^		Drysdale *et al.* ^11^	
	1252	Incidence	1239	Admitted	8927	Admitted	260	Admitted	324	Admitted	1273	injuries
	9/1/1998	8/31/2008	Jan-93	Dec-99	1/1/1987	12/31/1994	Feb-71	Aug-75	Jul-71	Jul-73	1970	
Bone	% of evaluations	% of fractures that were open	% of patients	% of fractures that were open	% of Patients	% of fractures that were open	% of patients	% of fractures that were open	% of Patients	% of fractures that were open	% of Patients	% of fractures that were open
Skull	8.23	15.53	2.99		8.92	Includes face fractures	11.15	NA	5.86		1.73	45.50
Face	13.66	1.75	1.53				7.93	NA	8.33		3.30	14.30
Spine Cervical	4.79		1.21		15.97	For all vertebrae	0.38	NA	4.94	For all vertebrae	1.65	0.00
Thoracic	3.83		7.02				0.38	NA			For all vertebrae	
Lumbar	7.59		1.37				4.23	NA				
Ribs/ Sternum/Flail Chest	16.93		8.23				8.08	NA	7.10		1.73	0.00
Clavicle	8.79	2.73	7.18				2.69	14.29	4.94		3.85	8.20
Scapula	4.31		1.86								0.63	0.00
Humerus	3.12	23.08	5.08				3.08	25.00	2.47		0.63	12.50
Radius/Ulna	10.14	20.47	3.39		15.32		11.15	13.79	8.95		4.71	13.31
Hand/Wrist	8.63	8.33	9.52				3.85	30.00			5.81	4.10
Acetabulum	2.72	2.94										
Pelvis	6.07	6.58	12.43								0.94	0.00
Femur	7.91	19.19	16.30		14.24		17.69	23.91			2.99	21.10
Patella	0.80	40.00									0.55	42.90
Tibia/Fibula	19.01	34.87	28.09		30.55		19.23	56.00			12.10	31.18
Foot	8.63	14.81	4.28				3.85	80.00			2.67	23.50
Additional demographic and outcome data	See Table I		Age 30.7 yr (22–36); 90.4% M; 10% no helmet; 6% dead		Age 25 yr (2–87); 91% M; LOS 2 (0-7900)		Age 22.41 yr (5–55); 90.4% M; 86.9% W, 3.5% B, 9.6% Hispanic; LOS 12.45 (1–136); 8% dead		LOS 39; 3% dead		1.4% dead	

Raw data used for the analysis in the study on epidemiology and patterns of musculoskeletal injuries The study raw data were retrieved from a prospectively collected injury database established at our Level I trauma center in accordance with the study inclusion and exclusion criteria. The raw data were subsequently reviewed for consistency and completeness prior to statistical analysis.

## Discussion

Many epidemiological studies have addressed motorcycle crash injuries, in particular the effect of helmet use ^3, 5, 6, 9, 25^. There has been a relative paucity, however, of new information regarding orthopedic motorcycle crash injuries in USA over the last 30 years. Unfortunately, during this period motorcycle crashes continued to be a major cause of accident-related morbidity and mortality. It has been reported that in 2007 motorcyclists were 37 times more likely to die in a motor vehicle traffic crash than passenger car occupants, and nine times more likely to be injured ^20^. Whereas car and truck fatality rates have decreased for the past 6 and 3 years, respectively, 2008 marked the eleventh consecutive year in which the annual motorcycle fatality rate increased ^19^. Yet, to our knowledge, the most recent epidemiological studies on orthopedic motorcycle crash injuries in the USA were by Bried *et al.* ^6^ in 1987 and Peek *et al.* ^22^ in 1994.

According to our data, the rates of skull, face, spine, rib, and pelvis fractures have increased in the USA motorcycle crashes compared with historical reports ^9, 11, 28^. The rates of skull and face fractures in our study are substantially higher than in recent European studies ( Table VI), whereas the rates of spine and pelvis fractures have remained similar. The higher incidence of some of the injuries documented in our study may be attributed to better injury detection. In the USA, whole-body computed tomography has become a routine trauma screening tool ^24^. Moreover, European riders are more likely to wear a helmet ^2^, and their lower incidence of skull and face fractures and death might be attributed to this protective device. The protective nature of helmets has been described in a number of studies ^13, 16^.

In contrast to Ankarath *et al*’s ^2^ and Robertson *et al*’s ^23^ studies from 2002 that analyzed similar patient populations from the Yorkshire region of the United Kingdom from the mid to late 1900s and Kupferschmid *et al.* ^15^ study from 1989, we found lumbar spine fractures to be more common than thoracic. This is in agreement with Goslar *et al.*’s ^12^ study (2008) that looked at spine fractures in motorcycle accident victims in Arizona. However, the incidence of facial fractures in the present study was nearly twice that reported by Kraus *et al.* (13.66% vs. 7.10%) in a study using early 1990s data ^14^, when routine computed tomography was not as common a procedure.

Injuries and death among older motorcyclists has recently become a focus topic of motorcycle crash studies. It has been reported that the age of motorcyclists has been increasing ^7^. The Motorcycle Industry Council found the mean age of motorcycle ownership to rise from 33.1 years in 1998 to 40.2 years in 2003 ^17^. Our study of patients aged 4 to 83 years (mean 36 years) joins other studies of motorcycle crash victims that have shown increased risk for a longer hospital stay, more severe injuries, and death among older patients. Brown *et al.*, using 1996–2005 National Trauma Databank statistics on 61,689 motorcycle operators involved in crashes, found the average age to increase steadily over the study period, from 33.9 years to 39.1 years ^7^. The fastest growing age group in relative frequency was that of 50 to 59 years, whereas the most rapidly declining was that of 20 to 29 years. Operators 40 years of age or older had higher ISS, LOS, intensive care unit LOS, and death rate compared with operators younger than 40 years. Brown *et al.* found significantly higher rates of severe thoracic and head injuries and rib fractures, as well as more complications and more than twice the rate of pre-existing comorbidities among motorcyclists aged 40 years or older ^7^. Whereas their data indicated no difference in helmet use between younger and older injured operators, we found older patients significantly less likely to use a helmet, with lack of helmeting linked to skull fracture and death ^3, 25^. In contrast, in a 1981 NHTSA treatise Hurt *et al.* described younger patients as less likely to wear a helmet ^13^.

Dischinger *et al.* found that older riders (40 years or older) have significantly higher incidence of thoracic injury, specifically multiple rib fractures ^10^. Talving *et al.* investigated the relation of age to injury type, distribution, and the severity of motorcycle crash patients in a Los Angeles County-wide trauma registry study ( *N* = 6,530) including data from 1995 to 1997 ^26^. Age was classified as 18 years or younger, 19 to 55 years, or older than 55 years. Older patients were significantly more likely to suffer severe trauma, severe head and chest injuries, and spinal fractures. Death was 3-fold higher in the oldest age group compared with the youngest.

The limitations of our study include its retrospective nature and the fact that it relies upon a trauma database. Although the data were collected prospectively, they were not specifically designed for this study. Orthopedic injuries were grouped by anatomic location as opposed to specific fracture type to control for small errors of injury classification (e.g., injury was classified as a radial shaft fracture as opposed to a Galeazzi or simple distal radius fracture). However, these injury classifications are similar to those employed in prior studies ^2, 6, 8, 9, 11, 22, 27, 28^, and many of those study data were also derived from existing databases ^2, 4, 12^.

Although the motorcycle fatality rate continues to increase, we did not see a similar increase in in-hospital mortality rate, indicating that more of the involved participants are likely being declared dead at the scene. The increase in the incidence of skull, spine, and face injuries noted in the study may be attributed to better injury detection (routine CT and/or MRI). The higher incidence of USA skull and facial fractures, as compared to other countries, may also be attributed to differences in compulsory helmet wearing laws. Older MI patients are a higher risk for serious injury and mortality, and this is further potentiated by not wearing a helmet.

## Data availability

***Figshare:*** Raw data used for the analysis in the study on epidemiology and patterns of musculoskeletal injuries. Doi: http://dx.doi.org/10.6084/m9.figshare.1402186 ^29^
