# Supplementary material for: Epidemiology and patterns of musculoskeletal motorcycle injuries in the USA
Source: F1000Res. 2015 May 12;4:114. [Version 1] doi: 10.12688/f1000research.4995.1 (PMC4536615; doi:10.12688/f1000research.4995.1)
Supplement: Peer review history

# Peer review: Referee response for version 1

**DOI:** 10.5256/f1000research.5332.r8631

**Published:** 2015-05-28

**Authors:** Arvind D. Nana

**Affiliations:** 1 Department of Orthopaedic Surgery, University of North Texas Health Science Center, Fort Worth, TX, USA

Good paper. Injury details are well captured. For instance, this study did a better job of capturing acetabulum, pelvis, patella, and scapula fractures compared to previous studies. The authors did identify dislocations in this patient population, and I wish they had discussed that further in the paper.

I have read this submission. I believe that I have an appropriate level of expertise to confirm that it is of an acceptable scientific standard.

# Peer review: Referee response for version 1

**DOI:** 10.5256/f1000research.5332.r8630

**Published:** 2015-05-21

**Authors:** Charles A. Reitman

**Affiliations:** 1 Department of Orthopaedic Surgery, Baylor College of Medicine, Houston, TX, USA

Well written study on relevant topic.

Weaknesses are discussed by the authors. Conclusions supported by the data.

I support indexation.

I have read this submission. I believe that I have an appropriate level of expertise to confirm that it is of an acceptable scientific standard.
